# Bioactive Pregnane Steroids from a South China Sea Gorgonian *Carijoa* sp.

**DOI:** 10.3390/molecules18033458

**Published:** 2013-03-15

**Authors:** Hong-Ying Zhao, Chang-Lun Shao, Zhi-Yong Li, Lei Han, Fei Cao, Chang-Yun Wang

**Affiliations:** 1 Key Laboratory of Marine Drugs, The Ministry of Education of China, School of Medicine and Pharmacy, Ocean University of China, Qingdao 266003, China; E-Mails: zhaohongying786@126.com (H.-Y.Z.); shaochanglun@163.com (C.-L.S.); leihan09@gmail.com (L.H.); caofei542927001@163.com (F.C.); 2 Marine Biotechnology Laboratory, State Key Laboratory of Microbial Metabolism, School of Life Sciences & Biotechnology, Shanghai Jiao Tong University, Shanghai 200240, China

**Keywords:** gorgonian coral, *Carijoa* sp., pregnane steroid, cytotoxicity, antibacterial activity

## Abstract

A new pregnane steroid, **1**, and three known analogues **2**–**4**, have been isolated from a gorgonian *Carijoa* sp. collected from the South China Sea. The planar structure and relative configuration of **1** were elucidated from comprehensive spectroscopic data. Its absolute configuration was determined by application of the modified Mosher method. Compounds **1**, **3** and **4** exhibited cytotoxicity against the human hepatoma cell line Bel-7402, with IC_50_ values of 9.33, 11.02 and 18.68 µM, respectively. Additionally, compound **1** exhibited promising antibacterial activity against *Pseudomona puido*, with a MIC value of 31 nM, which is approximately 5-fold more potent than ciprofloxacin (MIC = 156 nM).

## 1. Introduction

**Figure 1:**
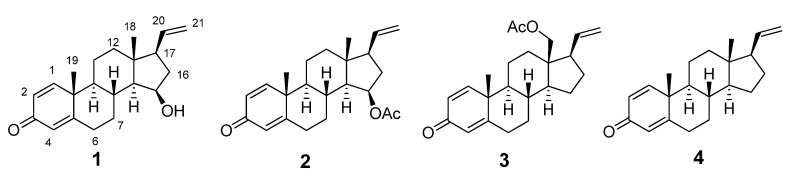
Chemical structures of compounds **1**–**4**.

Steroidal compounds from marine organisms possess a wide array of unusual structures. Among them, pregnane steroids characterized by an uncommon vinyl side chain represent a minor group of metabolites, and octocorals appear to be their most prolific source [1,2,3]. Indeed, over 50 such compounds with vinyl side chains have been reported from the marine environment. These compounds have received much attention due to their multiple potent biological properties, including antibacterial [2], antiprotozoan [4,5] anti-inflammatory [6,7] and cytotoxic activities [7,8]. Recently, in the course of our investigation on new bioactive substances from corals collected from the South China Sea [9,10,11,12], the gorgonian *Carijoa* sp. attracted our attention because the EtOAc crude extract displayed significant toxicity toward the larvae of the brine shrimp *Artemia salina*, with a mortality rate of 88.6% at a concentration of 25 μg/mL. Chemical investigation on the active extract led to the isolation of four pregnanes with a 3-one framework: 15*β*-hydroxypregna-1,4,20-trien-3-one (**1**), 15*β*-acetoxypregna-1,4,20-trien-3-one (**2**) [13], 18-acetoxypregna-1,4,20-trien-3-one (**3**) [13], and pregna-1,4,20-trien-3-one (**4**) [14,15] (Figure 1). Their structures were elucidated by NMR spectroscopic methods and comparison with data previously reported in the literature. Among these isolated compounds, **1** is a new pregnane steroid.

## 2. Results and Discussion

**Figure 2:**
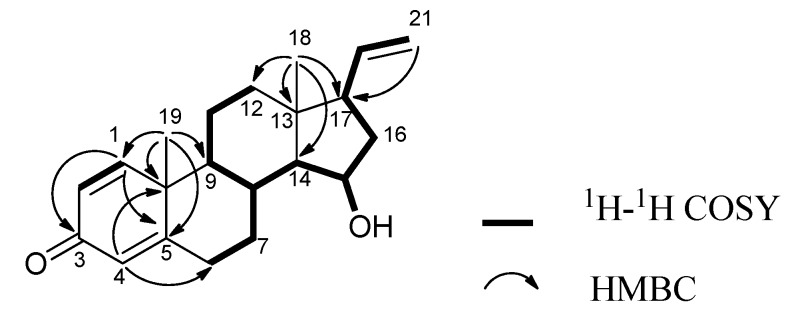
Key ^1^H–^1^H COSY and HMBC correlations for compound **1**.

**Figure 3:**
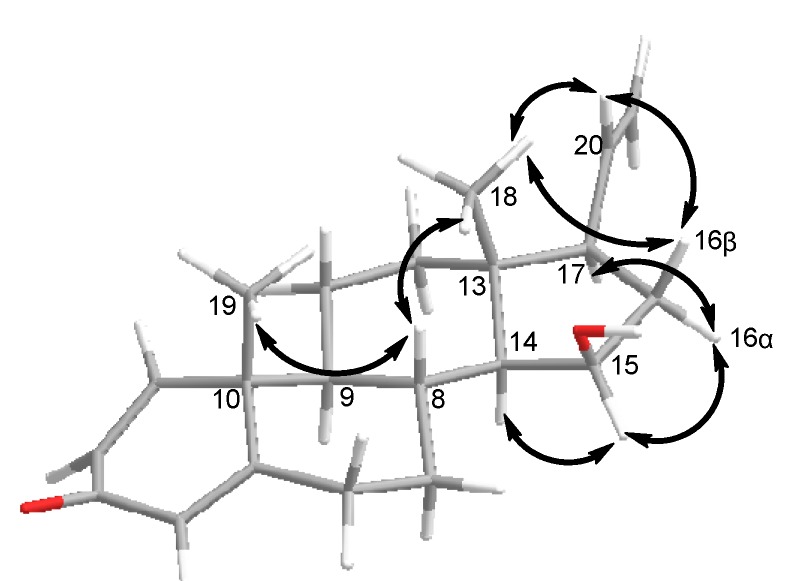
Selected NOESY correlations for compound **1**.

Compound **1** was obtained as pale yellow oil. Its molecular formula was determined as C_21_H_28_O_2_ by HRESIMS, suggesting eight degrees of unsaturation. Like **2**, the ^1^H-NMR spectrum of **1** (Table 1) showed the typical signals in the low-field region of a dienone system (H-1, H-2 and H-4) along with vinyl group (H-21a, H-21b and H-20) signals. The most obvious differences were the presence of one signal at *δ*_H_ 4.10 (m H-15) in **1** instead of the corresponding signal *δ*_H_ 5.10 (m, H-15) in **2**, and the disappearance of the methyl group signal at *δ*_H_ 2.05 (s, COCH_3_) in **1**. In the ^13^C-NMR spectra, the methine carbon signal of C-15 was shifted upfield (*δ*_C_ 69.0 in **1** *vs.* *δ*_C_ 73.7 in **2**) and the carbonyl carbon signal at *δ*_C_ 170.5, together with the acetyl methyl carbon signal at *δ*_C_ 21.6, had disappeared in **1**, which was the result of a hydroxy substituent at C-15 in **1**, instead of the acetoxy group in **2**. Furthermore, the position of the hydroxy group was confirmed as C-15 on the basis of the ^1^H–^1^H COSY correlation between H-15 (*δ*_H_ 4.10 m) and the hydroxyl proton (*δ*_H_ 4.45 d *J* = 4.0). The contiguous sequence of correlations from H-6 to H-12, and from H-8 to H-21 in the ^1^H–^1^H COSY spectrum (Figure 2) and the HMBC correlations (Figure 2) between H-1/C-3 and C-5, H-4/C-6 and C-10, H-21/C17, as well as from H-18 to C-12, C-13, C-14 and C-17, and from H-19 to C-1, C-5, C-9 and C-10 (Table 1) indicated that **1** has a 3-one pregnane skeleton similar to that of **2**, but with a hydroxy group at C-15. Treatment of **2** with NaOH in the presence of ethanol afforded **1** as the major product, which further confirmed the structure of **1**. Because the relative configurations of **2** have been previously established, the chemical conversion from **2** allowed the determination of *β*-orientation of hydroxy group at C-15 in **1**, and the NOESY correlations between H-14/H-15, H-15/H-16*α* and H-16*α*/H-17, as well as H-18/H-16*β*, H-16*β*/H-20 and H-20/H-18, also confirmed the *β*-stereochemistry of the hydroxy group at C-15. This configuration was also confirmed by the correlations between H-19/H-8 and H-8/H-18 in the NOESY spectrum (Figure 3).

**Table 1:** ^1^H and ^13^C-NMR data for compound **1** (400 and 100 MHz, in DMSO, ppm, *J*/Hz).

Positon	*δ*_H_	*δ*_C_	HMBC
1	7.21 d (10.1)	156.9	C-3, C-5, C-9, C-10, C-19
2	6.11 dd (10.1, 1.9)	127.1	C-10
3	–	185.3	
4	6.00 s	123.3	C-6, C-10
5	–	170.1	
6	2.48 m2.33 m	32.4	C-5
7	2.27 m0.98 m	32.7	
8	1.94 m	31.7	
9	1.05 m	53.1	C-19
10	–	43.8	
11	1.68 m1.61 m	22.3	
12	1.57 m1.01 m	38.3	C-18
13	–	43.3	
14	0.78 dd (11.1, 5.8)	59.0	C-8, C-13, C-18
15	4.10 m	69.0	
16	2.18 m ( *α* )	39.7	C-13
	1.51 m ( *β* )		
17	1.85 m	55.0	
18	0.87 s	15.7	C-12, C-13, C-14, C-17
19	1.22 s	18.9	C-1, C-5, C-9, C-10
20	5.79 ddd(17.3, 10.4, 7.4)	139.6	
21	4.98 dd (10.4, 1.7)	115.3	C-17
	4.96 dd (17.3, 1.7)		
15-OH	4.45 d (4.0)		

**Figure 4:**
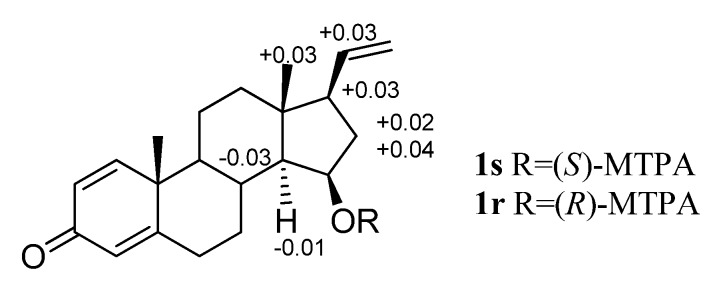
Values of Δ*δ*_H(*S*–*R*)_ (measured in CDCl_3_) of the MTPA esters of compound **1**.

The absolute configuration of **1** was established by the modified Mosher method [16]. Treatment of **1** with (*S*)-(+)-*α*- and (*R*)-(–)-*α*-methoxy-*α*-(trifluoromethyl)phenylacetyl chloride (MTPA-Cl) gave the corresponding (*R*)- and (*S*)-MTPA esters **1r** and **1s**, respectively. The ^1^H-NMR signals of the two MTPA esters were assigned on the basis of their ^1^H–^1^H COSY spectra. The Δ*δ*_H(*S*__–*R*)_ values were then calculated (Figure 4). Following the literature [16], the results indicated that the absolute configuration of C-15 was *R*. Therefore the absolute configurations at C-8, C-9, C-10, C-13, C-14 and C-17 in **1** were assigned as *R*, *S*, *S*, *R*, *S* and *R*, respectively. On the basis of the above evidence, the chemical conversion from **2** to **1** allowed the determination of the 8*R*,9*S*,10*S*,13*R*,14*S*,15*R*,17*R* configurations for **2** as the first report of its absolute configuration.

Compound **2** differed from **1** only in the acetate group at C-15. Actually we used EtOAc for their extraction and isolation, but even though **1** was dissolved in EtOAc and stirred at 40 °C for one week, **2** was not detected in solution, therefore, it seems very unlikely that **2** is an artifact of the isolation procedure. Furthermore, **2** has also been isolated and confirmed as a new natural product from an Indopacific octocoral *Carijoa* sp. [13].

All the isolated compounds were evaluated for the cytotoxic activity against the human hepatoma Bel-7402 and human normal embryonic lung fibroblast MRC-5 cell lines. Compounds **1**, **3** and **4** showed cytotoxicity to Bel-7402, with IC_50_ values of 9.33, 11.02 and 18.68 µM, respectively. Compound **2** exhibited moderate cytotoxicity against Bel-7402, with an IC_50_ = 73.47 µM. Additionally, compounds **1**–**4** showed weak cytotoxicity against MRC-5.

The antibacterial activity of compounds **1**–**4** was further evaluated *in vitro* against a panel of pathogenic bacteria [17], including *Bacillus cereus*, *Tetragenococcus halophilus*, *Staphylococcus albus*, *Staphylococcus aureus*, *Escherichia coli*, *Pseudomonas putida*, *Nocardia brasiliensis*, and *Vibrio parahaemolyticus* (Table 2). Compounds **1** and **2** showed a broad spectrum of antibacterial activity. Compound **1** showed significant antibacterial activity against *S. aureus*, *S. albus*, *E. coli*, *V. parahaemolyticus* and *N. brasiliensis*, with MIC values of 0.063, 1.00, 1.00, 4.00, and 0.500 μM, respectively, and exhibited promising inhibitory activity against *P. putida*, with an MIC value of 31 nM, which was approximately 5-fold more potent than that of ciprofloxacin (MIC = 156 nM). Compound **2** inhibited seven pathogenic bacteria, but not *S. aureus*, and was especially active against *T. halophilus*, with a MIC value of 312 nM. Additionally, Compound **3** showed antibacterial activity against *B. cereus*, *S. aureus* and *T. halophilus* with MIC values of 2.50, 0.156, and 1.25 μM, respectively.

**Table 2:** Tests of MIC (μM) for compounds **1**–**4** against eight bacterial strains.

Strains	Compounds				
	1	2	3	4	Ciprofloxacin
*B. cereus*	>64.0	5.00	2.50	>50.0	0.078
*S. aureus*	0.063	>50.0	0.156	>50.0	0.019
*S. albus*	1.00	2.50	>50.0	>50.0	0.312
*T*. *halophilus*	>64	0.312	1.25	>50.0	0.019
*E. coli*	1.00	2.50	>50.0	>50.0	0.625
*P. putida*	0.031	1.25	>50.0	>50.0	0.156
*V. parahaemolyticus*	4.00	50.0	>50.0	>50.0	2.50
*N*. *brasiliensis*	0.500	1.25	>50.0	>50.0	0.078

## 3. Experimental

### 3.1. General Procedures

Optical rotations were measured on a JASCO P-2000 digital polarimeter at room temperature. IR spectra were recorded on a Nicolet 6700 spectrometer. UV spectra were measured on a Nicolet Evolution 300 spectrophotometer. ESI-MS and HR-ESI-MS were recorded on a Q-Tof Premier LC mass spectrometer. NMR spectra were recorded on an AVANCE III 400 (400 MHz for ^1^H-NMR and 100 MHz for ^13^C-NMR) spectrometer and a JEOL Eclips-600 spectrometer. Chemical shifts (*δ*) were reported in ppm relative to an internal TMS standard, and coupling constant (*J*) was reported in Hz. HPLC separation was performed in an Agilent 1200 semi-preparative HPLC system coupled with variable wavelength detector. A XDB-C_18_ preparative HPLC column (250 × 9.4 mm, 5 μm) was used. Analysis HPLC (Agilent 1200 HPLC system coupled with diode array detector) with XDB-C_18_ HPLC column (150 × 4.6 mm, 5 μm) was used. All solvents used were of analytical grade (Shanghai Chemical Plant, Shanghai, China). Silica gel (200–300 mesh; Qingdao Marine Chemical Group Co., Qingdao, China), octadecylsilyl (ODS) silica gel (45–60 mm; Merck KGaA, Darmstadt, Germany), and Sephadex LH-20 (Amersham Biosciences Inc., Piscataway, NJ, USA) were used for column chromatography. Precoated silica gel GF254 plates (Yantai Zifu Chemical Group Co., Yantai, China) were used for TLC analysis.

### 3.2. Animal Materials

Gorgonian *Carijoa* sp. (1.1 kg, wet weight) was collected off the coral reef of Weizhou Island in the South China Sea, China, in April 2011, and was identified by Dr. Xiu-Bao Li, South China Sea Institute of Oceanology, Chinese Academy of Science.

### 3.3. Extraction and Isolation

The frozen specimen was extracted with 95% EtOH (3 × 2000 mL) three times at room temperature for one week, and the solvent was evaporated *in vacuo*. The residue was partitioned in H_2_O (500 mL) and extracted with EtOAc three times (3 × 1,000 mL) at room temperature. The EtOAc extract was concentrated *in vacuo* to afford 8 g of EtOAc residue, which was subjected to column chromatography (CC) on silica gel, using petroleum ether (b.p. 60–90 °C)–EtOAc (from 20:1 to 0:10) as eluent. By combining the fractions according to TLC (GF_254_) monitoring, six fractions (Fr. 1−Fr. 6) were obtained. Fr. 2 (900 mg) was fractionated over silica gel CC eluted with petroleum ether−EtOAc gradients (from 25:1 to 3:1) to afford three sub-fractions (Fr.2.1−Fr.2.3). Repeated chromatography of Fr.2.3 using Sephadex LH-20 eluted with petroleum ether−CHCl_3_−MeOH (2:1:1) provided Fr.2.3.1−Fr.2.3.3, and then Fr.2.3.2 was purified by ODS CC eluted with MeOH to yield **2** (50 mg), and semi-preparative HPLC (MeOH:H_2_O = 80:20) to obtain **1** (4.3 mg), **3** (2.9 mg), and **4** (5.4 mg).

*15β-Hydroxypregna-1,4,20-trien-3-one* (**1**): pale yellow oil; [α]^26^_D_ −4° (*c* 0.4, CHCl_3_); UV (MeOH) λ_max_ (log*ε*) 246 (4.00) nm, IR (KBr) *ν*_max_ 3447, 2925, 2851, 1658, 1620, 1245, 910, 887 cm^−1^; ^1^H-NMR and ^13^C-NMR see Table 1; HRESIMS *m/z*: [M+H]^+^ 313.2168 (C_21_H_29_O_2_, calcd. 313.2168), [M+Na]^+^ 335.1987 (C_21_H_28_O_2_Na, calcd. 335.1987).

### 3.4. Hydrolysis of Compound ***2***

Compound **2** (20 mg) was dissolved in ethanol (2.0 mL), then NaOH (40 mg) was added, and the solution was allowed to stir at room temperature for 12 h. After that the mixture was neutralized with excess hydrochloric acid, water (10 mL) was added, and the solution was then extracted with EtOAc (10 mL × 3). The organic solvent was removed with a high-vacuum pump and the crude mixture was subjected to preparative HPLC to obtain **1** (15 mg).

### 3.5. Preparation of the (S)- and (R)-MTPA Ester Derivatives of Compound ***1***

4-(Dimethylamino)pyridine (2 mg) and (*R*)-(–)-*α*-methoxy-*α*-(trifluoromethyl)phenylacetyl chloride (MTPA-Cl, 10 μL) were added in a solution of **1** (1.5 mg) in pyridine (500 μL). The mixture was stirred at room temperature for 12 h. The reaction mixture was then passed through a disposable pipet packed with silica gel and eluted with petroleum ether–EtOAc (5:1) to give the (*S*)-Mosher ester **1s**. Treatment of **1** (1.5 mg) with (*S*)-MTPA-Cl (10 μL) as described above yielded the corresponding (*R*)-Mosher ester **1r**. Selected ^1^H-NMR (CDCl_3_, 600 MHz) of (*S*)-MTPA ester (**1s**): *δ*_H_ 7.42–7.56 (5H, Ph), 7.01 (1H, d, *J* = 10.1 Hz, H-1), 6.21 (1H, dd, *J* = 10.1, 1.9 Hz, H-2), 6.03 (1H, br t, H-4), 5.71(1H, ddd, *J* = 17.1, 10.3, 7.3 Hz, H-20), 5.34 (1H, m, H-15), 5.05 (1H, dd, *J* = 10.3, 1.5 Hz, H-21a), 4.99 (1H, dd, *J* = 17.1, 1.5 Hz, H-21b), 2.55 (1H, m, H-16a), 1.99 (1H, m, H-17), 1.68 (1H, m, H-8), 1.64 (1H, m, H-16b), 1.14 (3H, s, H_3_-19), 1.06 (1H, m, H-14), 0.72 (3H, s, H_3_-18); selected ^1^H-NMR (CDCl_3_, 600 MHz) of (*R*)-MTPA ester (**1r**): *δ*_H_ 7.43–7.55 (5H, Ph), 7.02 (1H, d, *J* = 10.1 Hz, H-1), 6.22 (1H, dd, *J* = 10.1, 1.9 Hz, H-2), 6.05 (1H, br t, H-4), 5.68 (1H, ddd, *J* = 17.1, 10.3, 7.3 Hz, H-20), 5.31 (1H, m, H-15), 5.03 (1H, dd, *J* = 10.3, 1.5 Hz, H-21a), 4.97 (1H, dd, *J* = 17.1, 1.5 Hz, H-21b), 2.53 (1H, m, H-16a), 1.96 (1H, m, H-17), 1.71 (1H, m, H-8), 1.60 (1H, m, H-16b), 1.17 (3H, s, H_3_-19), 1.07 (1H, m, H-14), 0.69 (3H, s, H_3_-18).

### 3.6. Brine Shrimp Lethality Assay

The growth inhibitory activity of the EtOAc extract was evaluated against the brine shrimp *Artemia salina* [18]. After incubation for 48 h, the brine shrimp lethality assay was performed on larvae of *A. salina* according to the published protocols [19]. Dimethyl sulfoxide (DMSO) was used as a negative control.

### 3.7. Cytotoxicity Test

The isolated compounds were screened for cytotoxic activity *in vitro* against the human hepatoma cell line Bel-7402 and the normal human embryonic lung fibroblast cell line MRC-5 using the tetrazolium (MTT) microculture method [20]. Well-growing carcinoma cells were collected and seeded in 96-well plates at 1 × 10^5^/mL density. When the cells anchored to the plates, the culture medium was replaced with fresh medium containing various concentrations of the compounds. Three duplicate wells were used for each sample. After incubation at 37 °C, 5% CO_2_ for 48 h, 50 μL MTT was added to each well for another 4 h incubation. Then, the MTT medium was discarded and warm dimethylsulfoxide (DMSO, 150 μL) was added. Absorbance was measured at 550 nm. Cisplatin was used as a positive control, and phosphate buffered saline was used as a blank control.

### 3.8. Antibacterial Activity Assay

Antibacterial activity was evaluated by the conventional broth dilution assay [17]. Eight bacterial strains: *B. cereus* (ACCC 11077), *S. albus* (ATCC 8799), *S. aureus* (ATCC 27154), *T. halophilus* (ATCC 13623), *E. coli* (ATCC 25922), *P. putida* (ATCC 17485), *N*. *brasiliensis* (ATCC 19019) and *V. parahaemolyticus* (ATCC 17802) were used, and ciprofloxacin was used as a positive control.

## 4. Conclusions

In this study, four pregnane steroids **1**–**4** were obtained from a gorgonian *Carijoa* sp., of which compound **1** is a new pregnane. The absolute configuration of **1** was determined by application of the modified Mosher method. In addition, the absolute configuration for **2** was also reported for the first time. Among steroids from corals, C_21_ pregnane and their glycosides characterized by the unusual vinyl side chain represent a minor group of metabolites, and most of them are substituted by glycosides and present hydroxy or acetyl groups at C-3 instead of uncommon 3-one dienone [9,21,22,23]. In this work, we found pregnanes all with rare 3-one dienones, suggesting some specific metabolic pathway in this species. 

## Supplementary Materials

Supplementary materials can be accessed at: http://www.mdpi.com/1420-3049/18/3/3458/s1.
