# Supplementary material for: Bioactive Pregnane Steroids from a South China Sea Gorgonian Carijoa sp
Source: Molecules. 2013 Mar 15;18(3):3458–66. doi: 10.3390/molecules18033458 (PMC6270022; doi:10.3390/molecules18033458)

Supplementary Materials

Figure S1. ^{1}H-NMR spectrum (400 MHz, DMSO) of compound **1**.
Figure S2. Partial ^{1}H-NMR spectrum (400 MHz, DMSO) of compound **1**.
Figure S3. ^{13}C-NMR spectrum (100 MHz, DMSO) of compound **1**.
Figure S4. Partial ^{13}C-NMR spectrum (100 MHz, DMSO) of compound **1**.
Figure S5. HMQC spectrum of compound **1** in DMSO.
Figure S6. HMBC spectrum of compound **1** in DMSO.
Figure S7. ^{1}H–^{1}H COSY spectrum of compound **1** in DMSO.
Figure S8. NOESY spectrum of compound **1** in DMSO.
Figure S9. HRESI spectrum of compound **1**.
Figure S10. ^{1}H-NMR spectrum (400 MHz, $CDCl_3$) of compound **2**.
Figure S11. ^{13}C-NMR spectrum (400 MHz, $CDCl_3$) of compound **2**.
Figure S12. ^{1}H-NMR spectrum (400 MHz, DMSO) of compound **3**.
Figure S13. HRESI spectrum of compound **3**.
Figure S14. ^{1}H-NMR spectrum (400 MHz, DMSO) of compound **4**.
Figure S15. ^{1}H-NMR spectrum (600 MHz, $CDCl_3$) of compound **1**.
Figure S16. ^{1}H-NMR spectrum (600 MHz, $CDCl_3$) of compound **1s**.
Figure S17. ^{1}H–^{1}H COSY spectrum of compound **1s** in $CDCl_3$.
Figure S18. ESI-MS of compound **1s**.
Figure S19. ^{1}H-NMR spectrum (600 MHz, CDCl3) of compound **1r**.
Figure S20. ^{1}H–^{1}H COSY spectrum of compound **1r** in $CDCl_3$.
Figure S21. ESI-MS of compound **1r**.

Figure S1. ^{1}H-NMR spectrum (400 MHz, DMSO) of compound **1**.

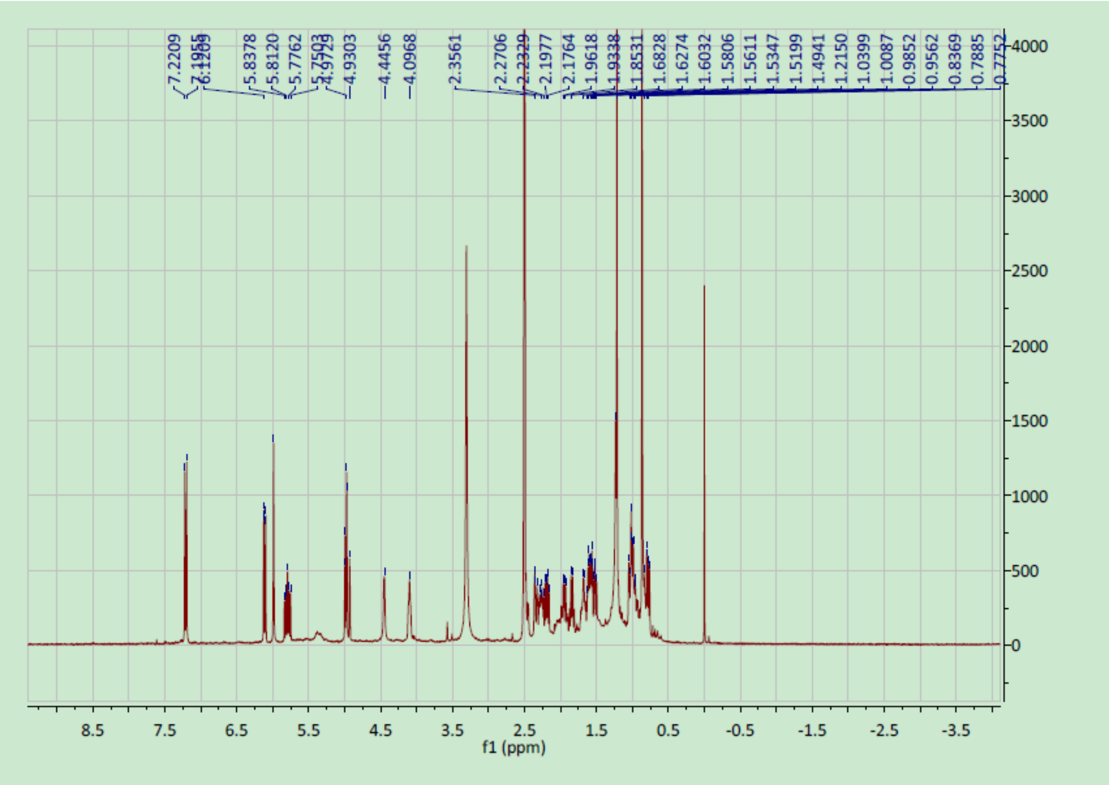

Figure S2. Partial ^{1}H-NMR spectrum (400 MHz, DMSO) of compound **1**.

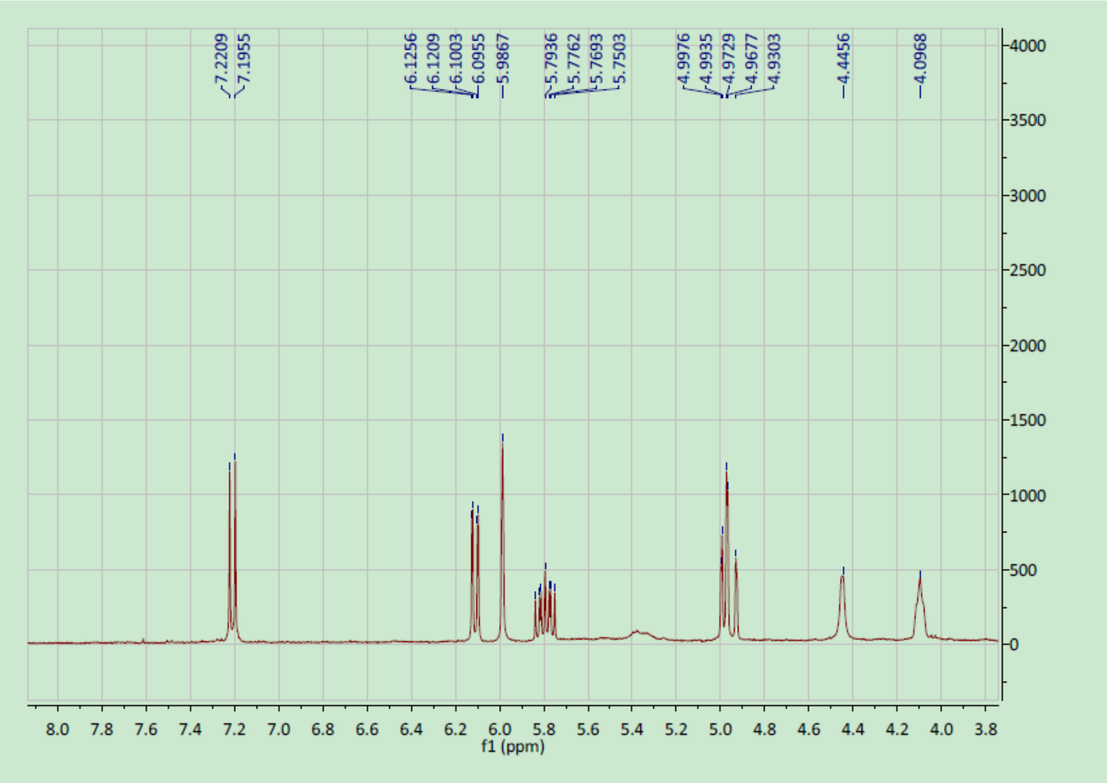

Figure S3. ^{13}C-NMR spectrum (100 MHz, DMSO) of compound **1**.

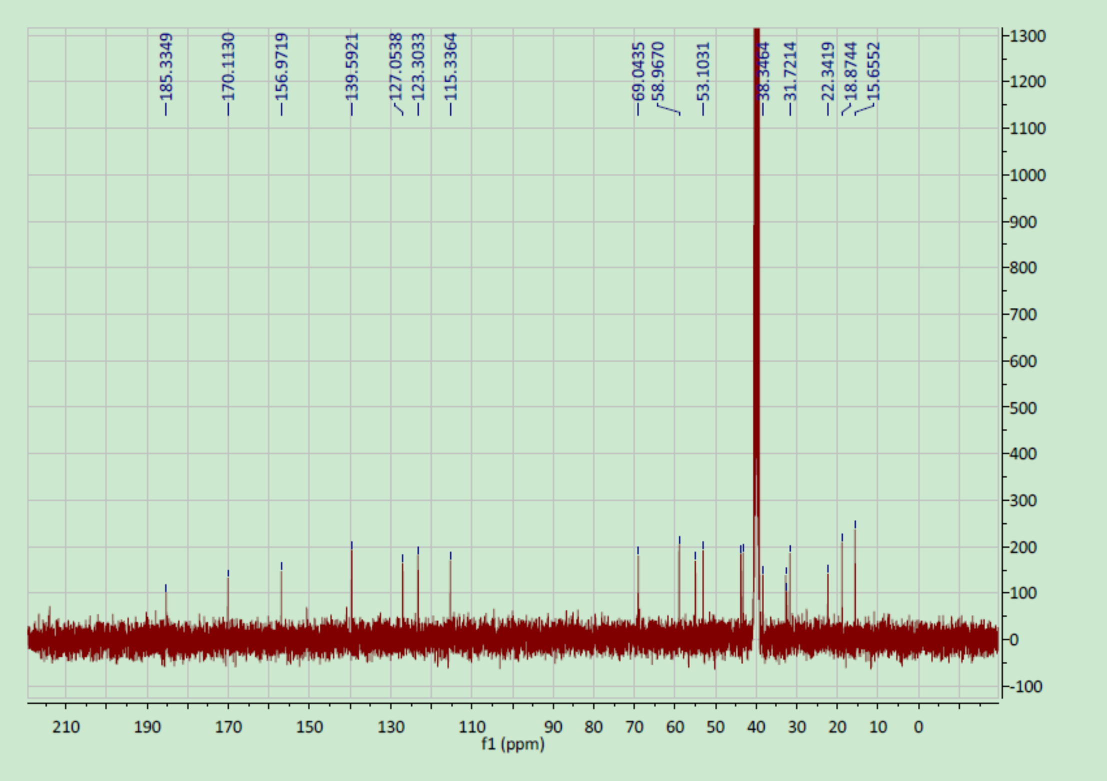

Figure S4. Partial ^{13}C-NMR spectrum (100 MHz, DMSO) of compound **1**.

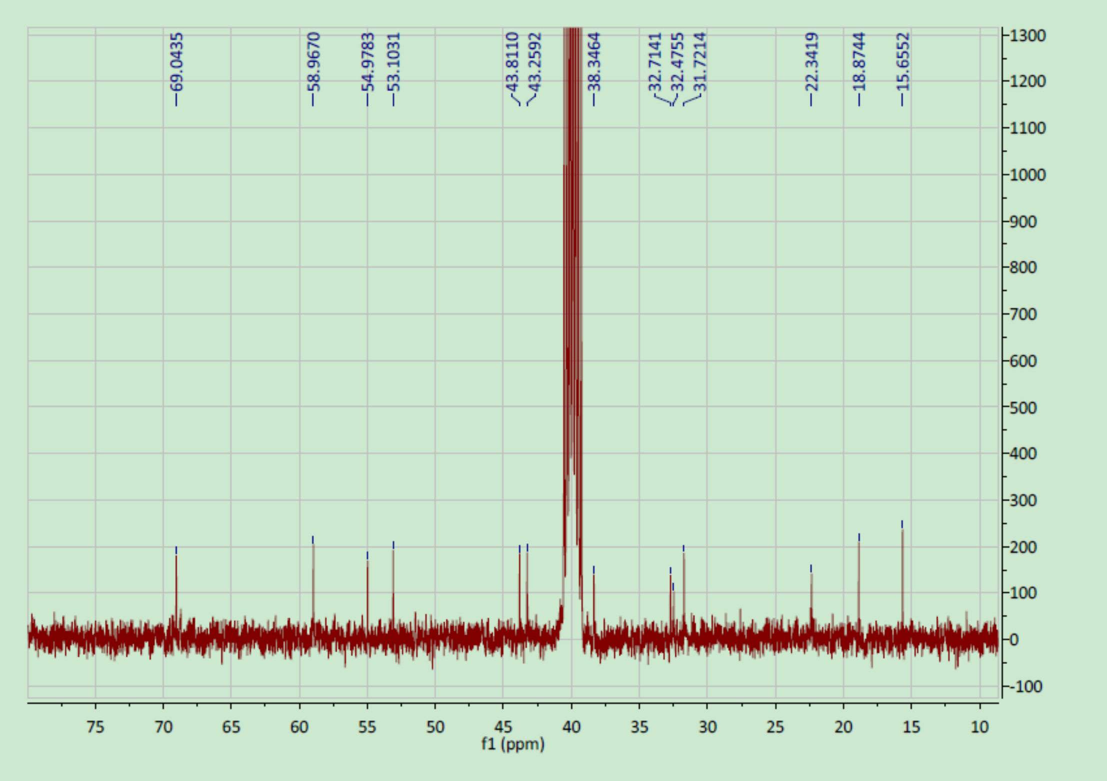

Figure S5. HMQC spectrum of compound **1** in DMSO.

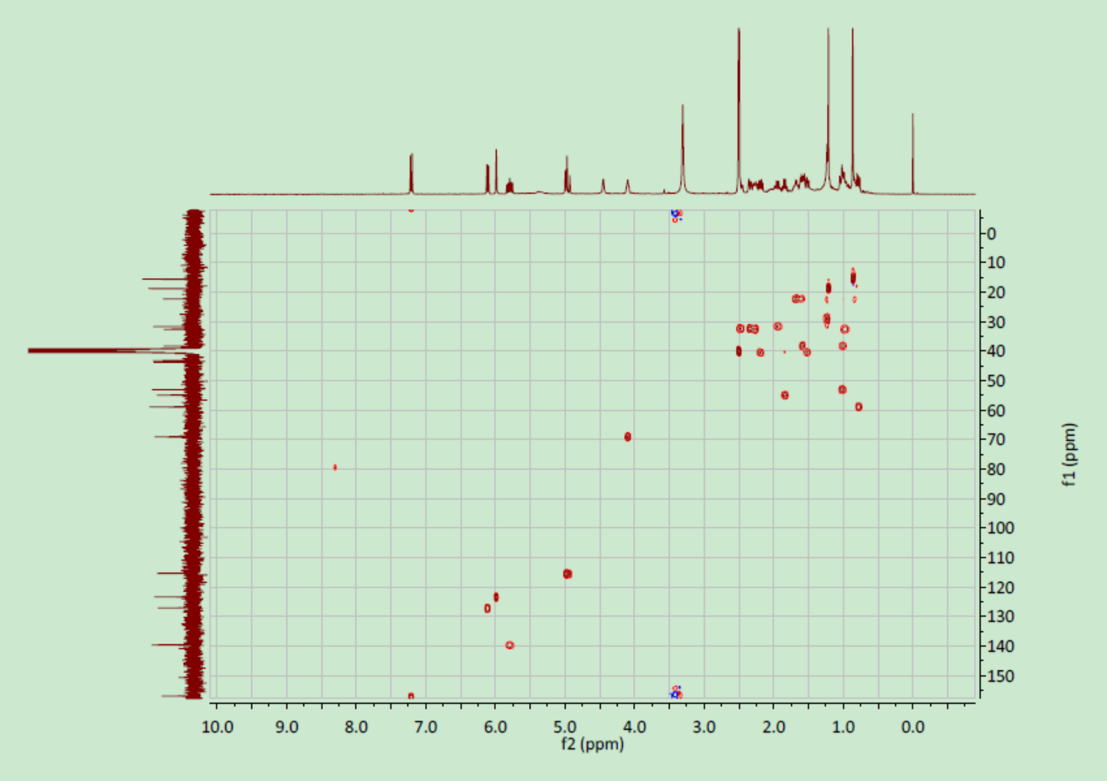

Figure S6. HMBC spectrum of compound **1** in DMSO.

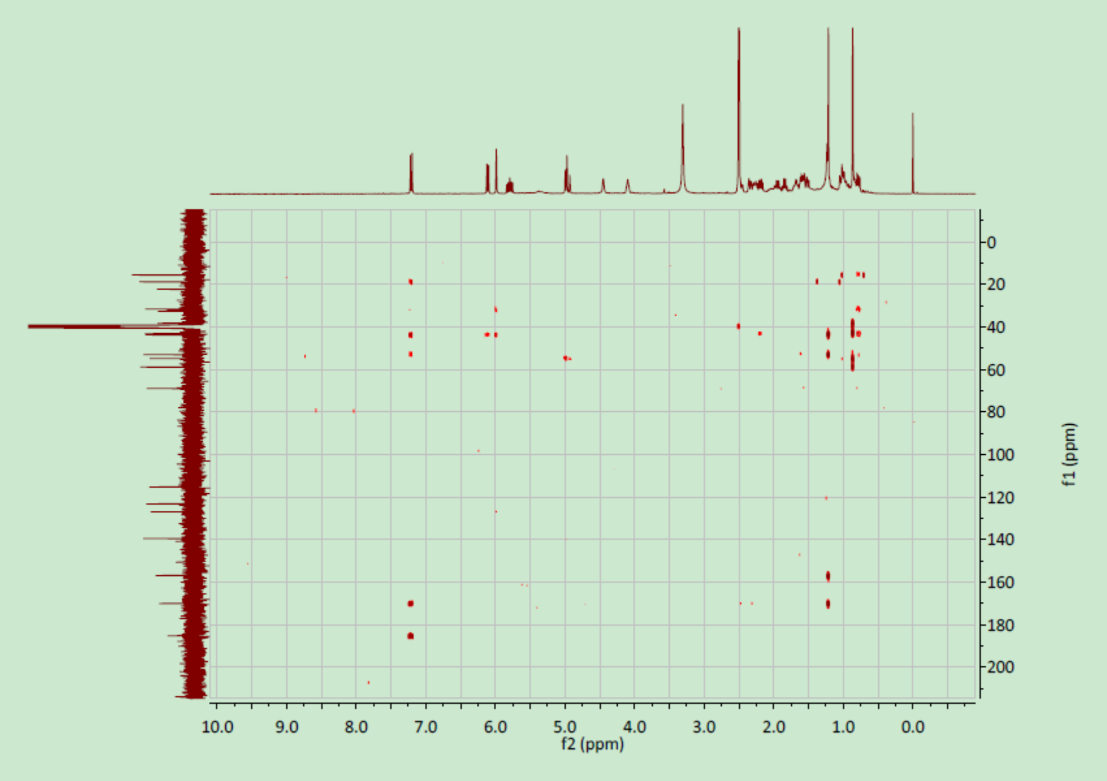

Figure S7. ^{1}H–^{1}H COSY spectrum of compound **1** in DMSO.

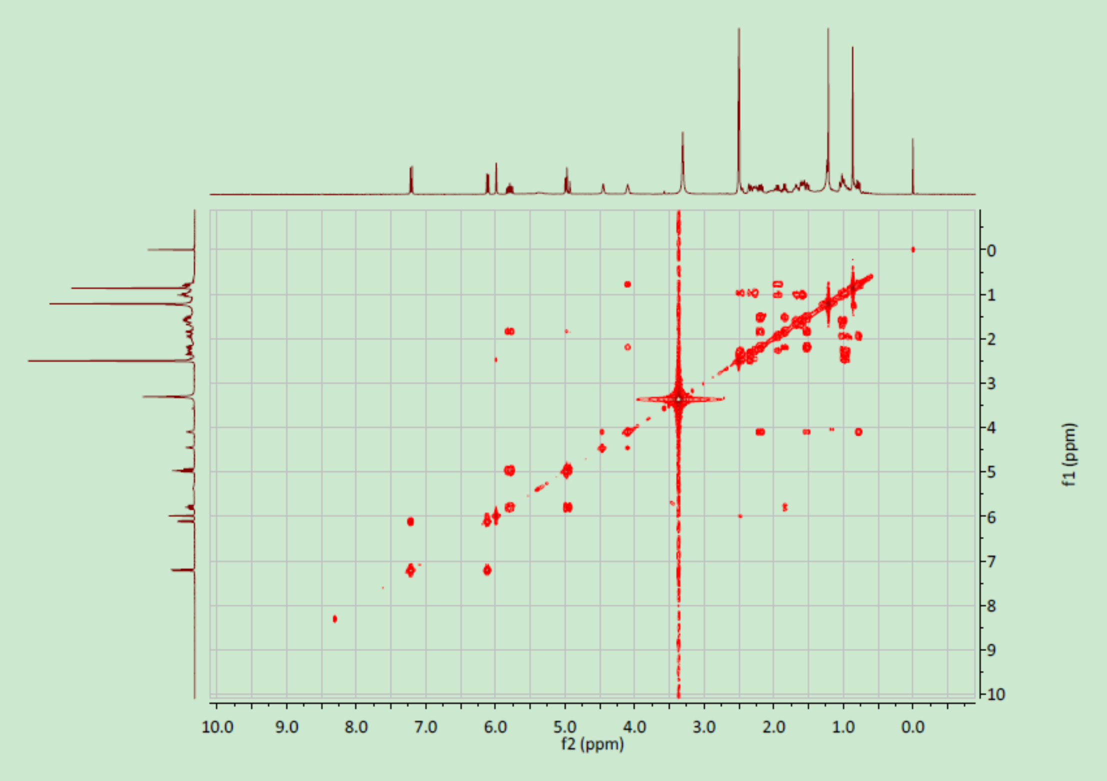

Figure S8. NOESY spectrum of compound **1** in DMSO.

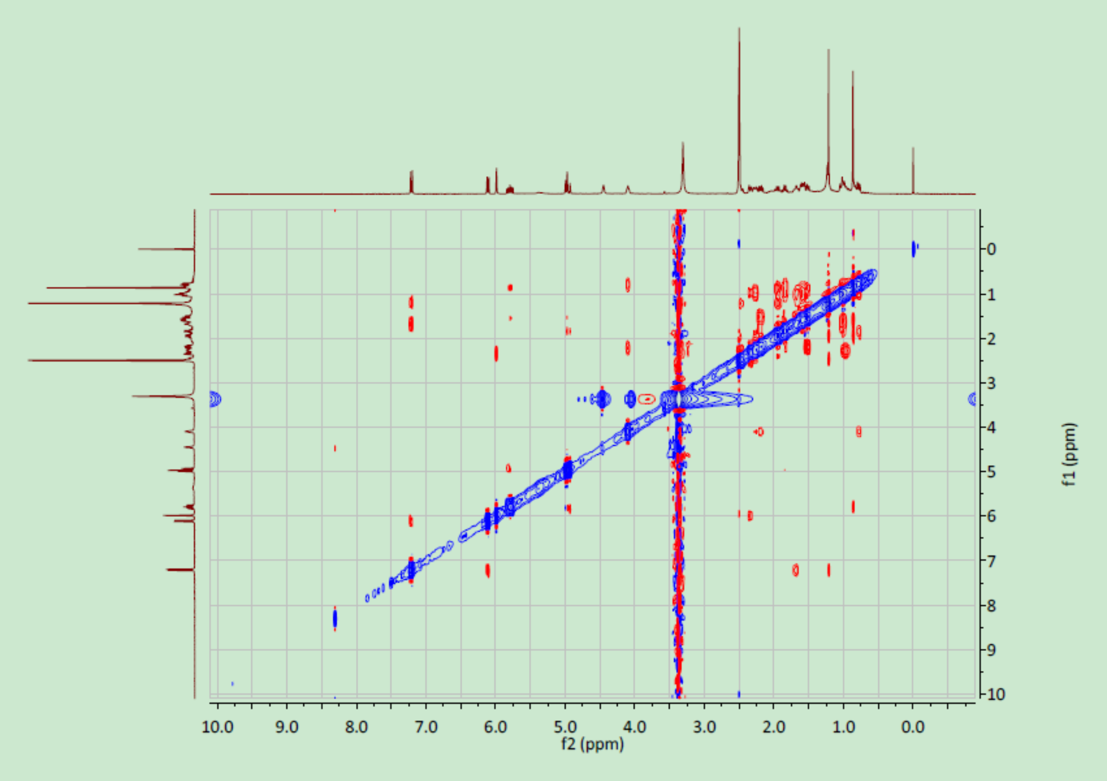

Figure S9. HRESI spectrum of compound **1**.

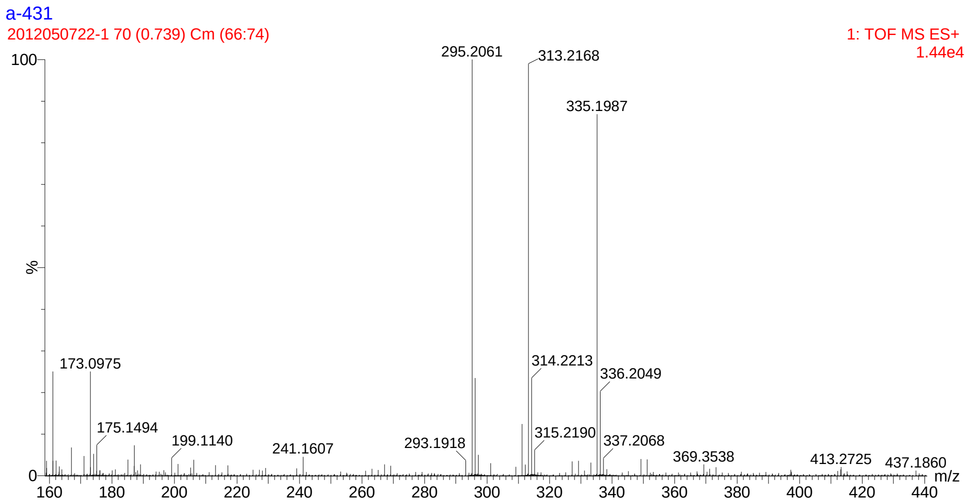

Figure S10. ^{1}H-NMR spectrum (400 MHz, $CDCl_3$) of compound **2**.

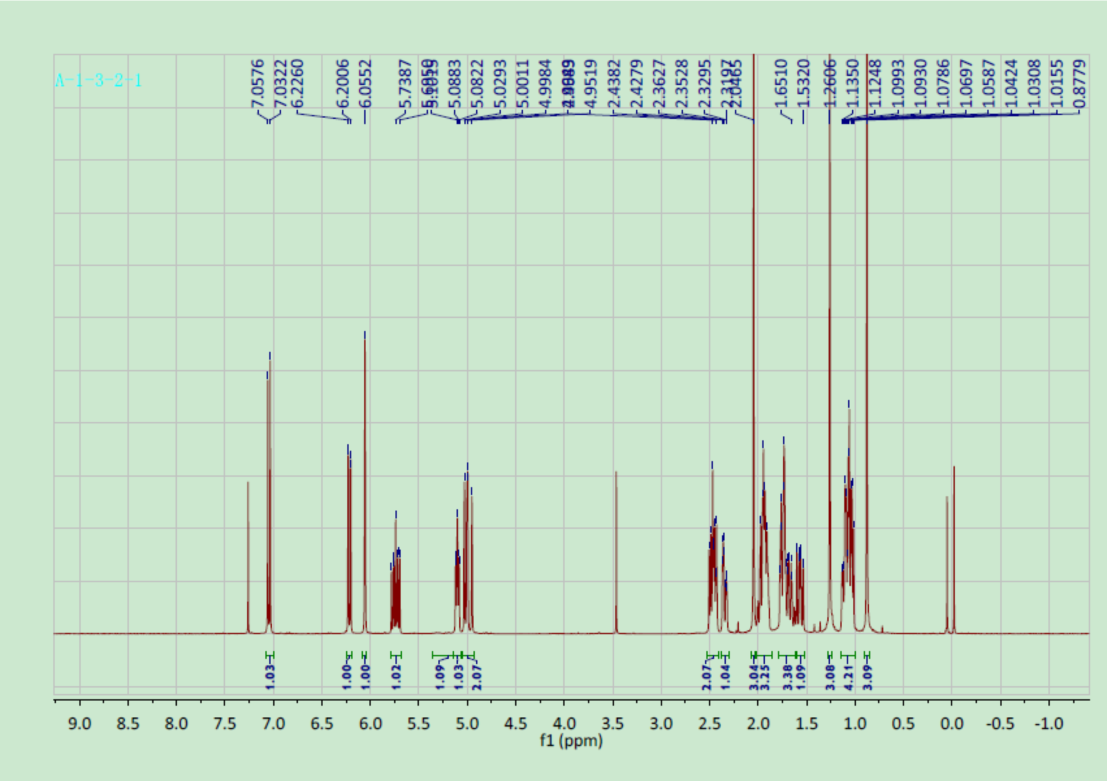

Figure S11. ^{13}C-NMR spectrum (400 MHz, $CDCl_3$) of compound **2**.

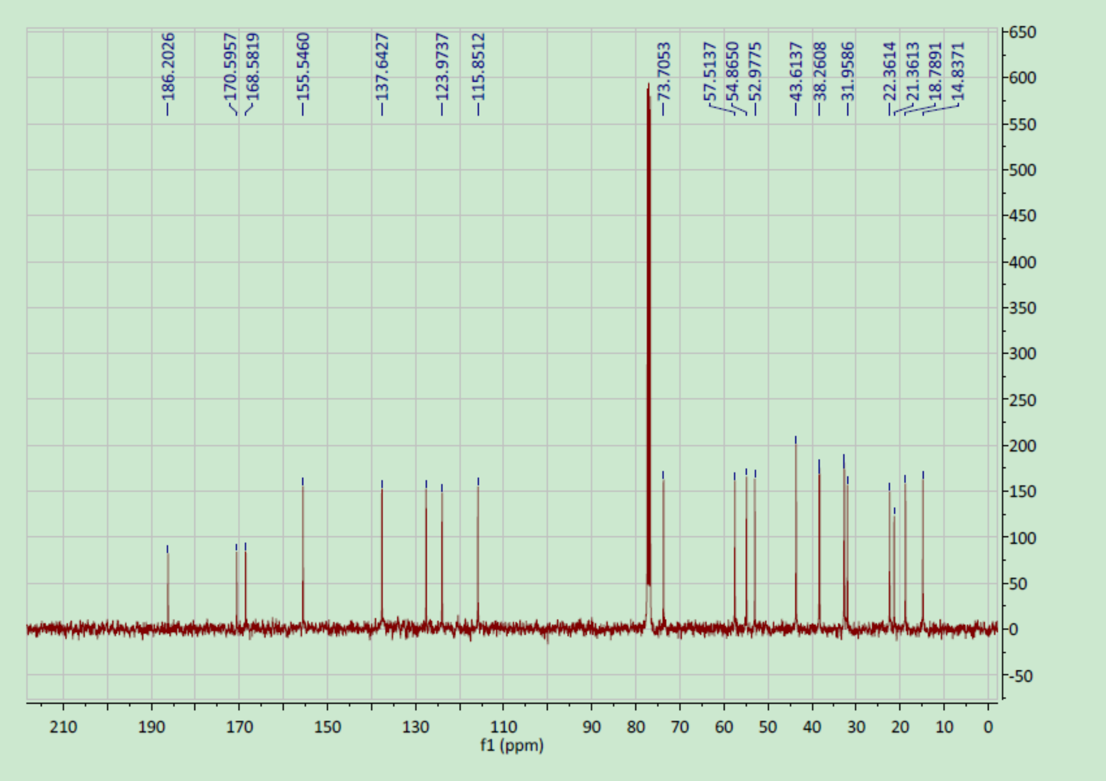

Figure S12. ^{1}H-NMR spectrum (400 MHz, DMSO) of compound **3**.

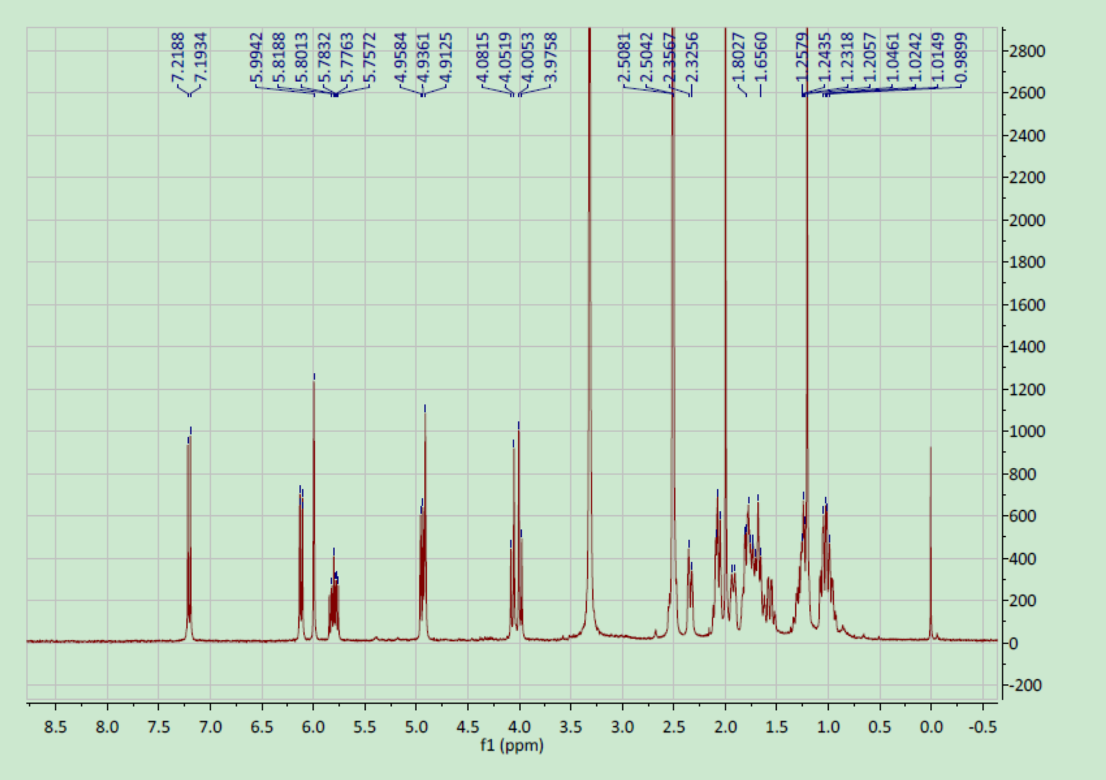

Figure S13. HRESI spectrum of compound **3**.

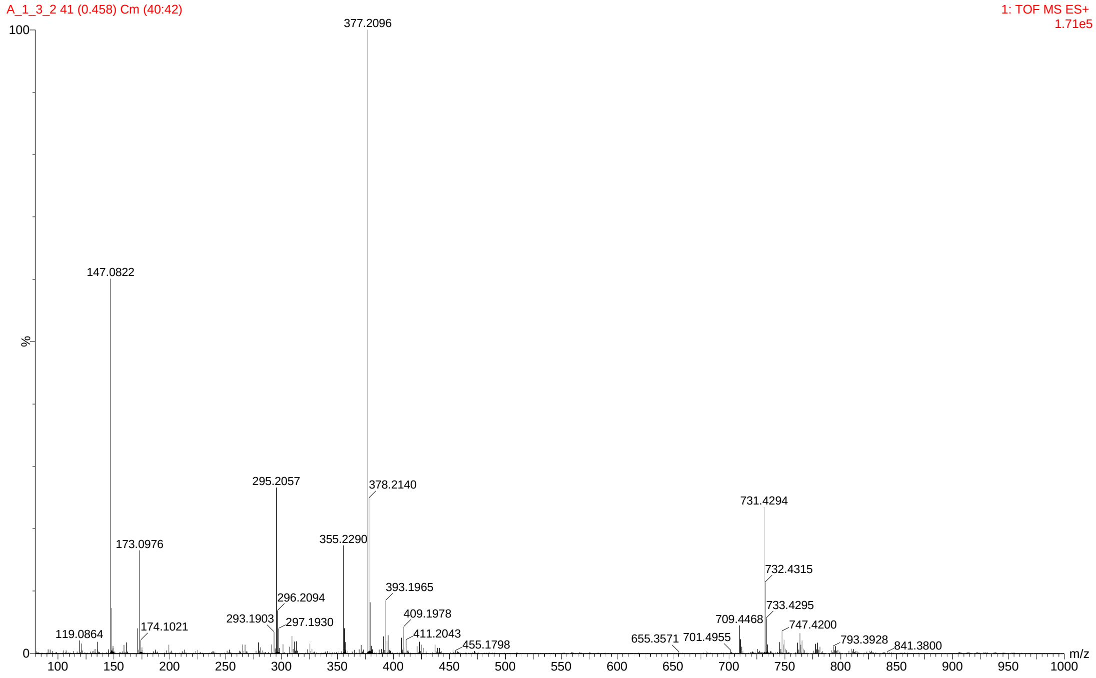

Figure S14. ^{1}H-NMR spectrum (400 MHz, DMSO) of compound **4**.

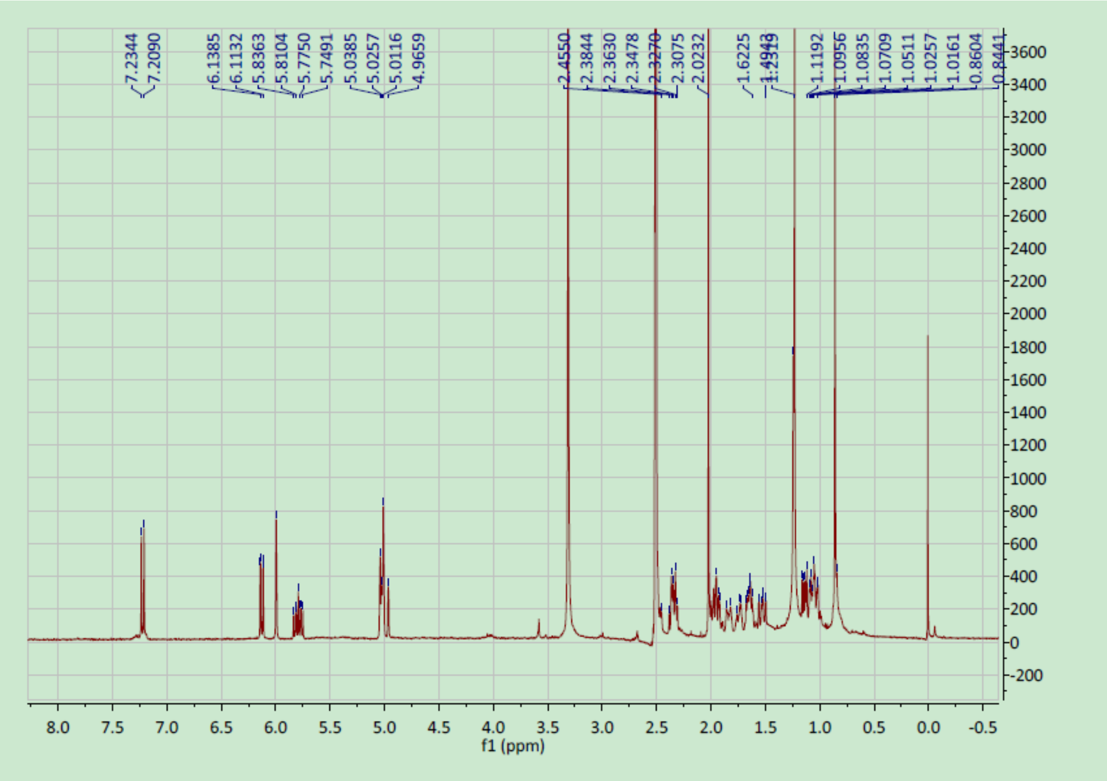

Figure S15. ^{1}H-NMR spectrum (600 MHz, $CDCl_3$) of compound **1**.

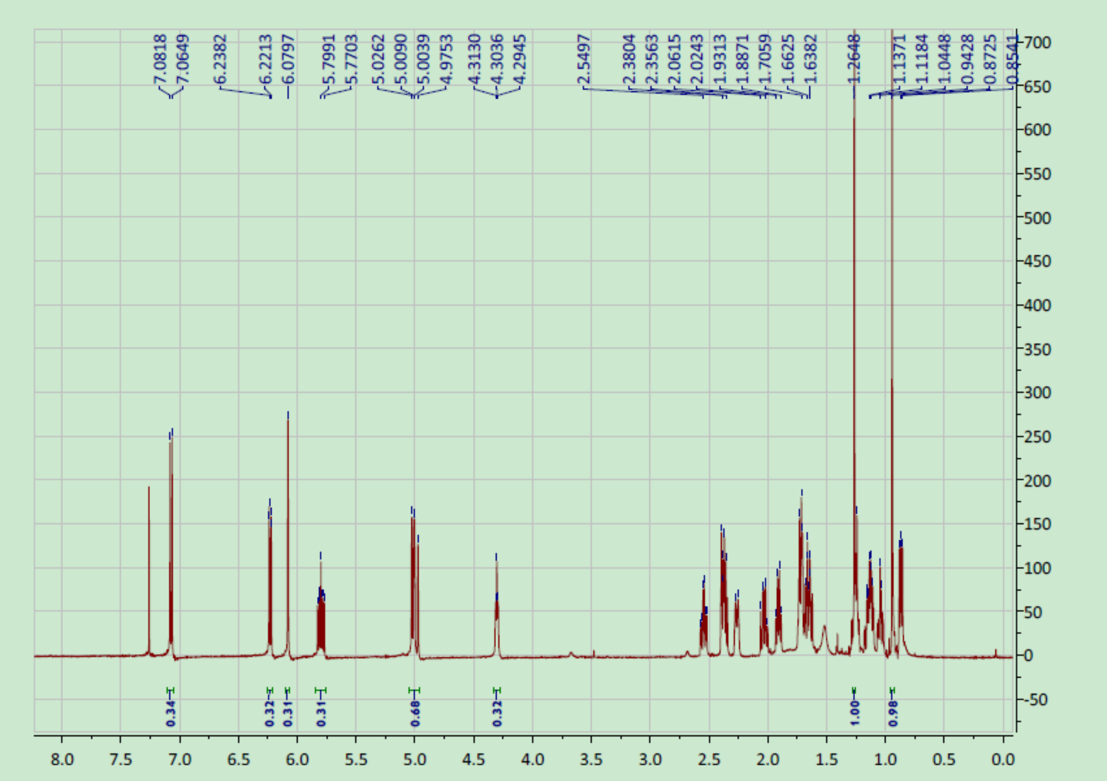

Figure S16. ^{1}H-NMR spectrum (600 MHz, $CDCl_3$) of compound **1s**.

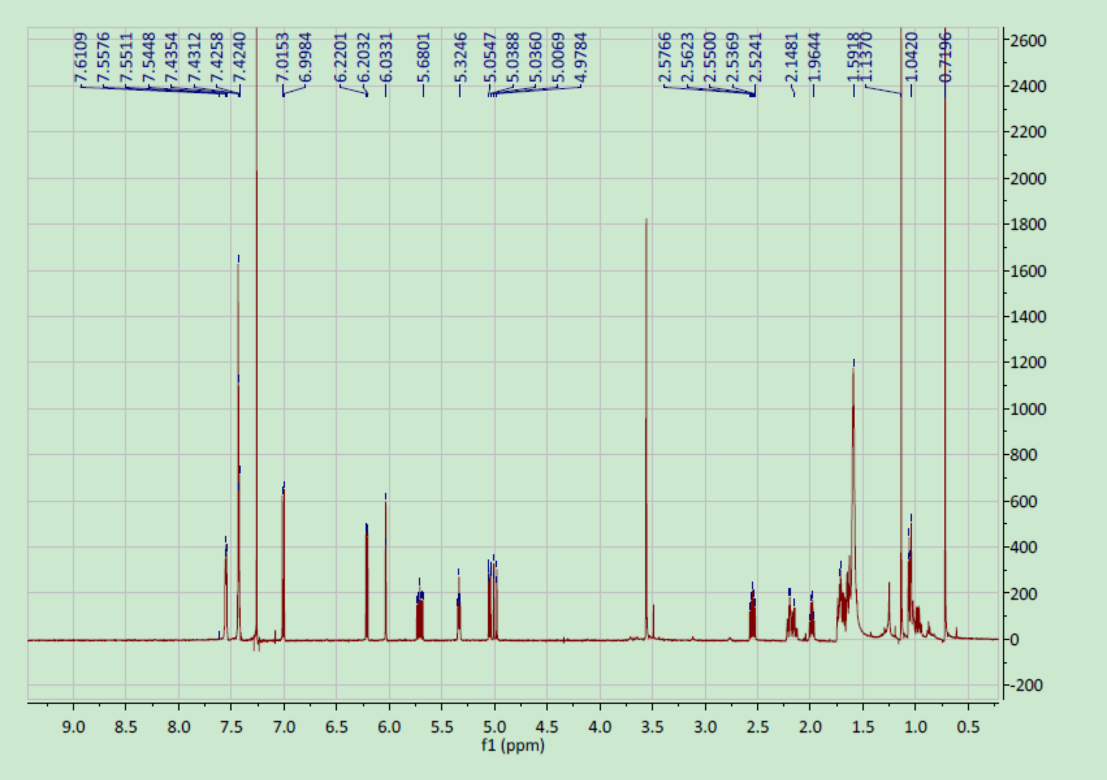

Figure S17. ^{1}H–^{1}H COSY spectrum of compound **1s** in $CDCl_3$.

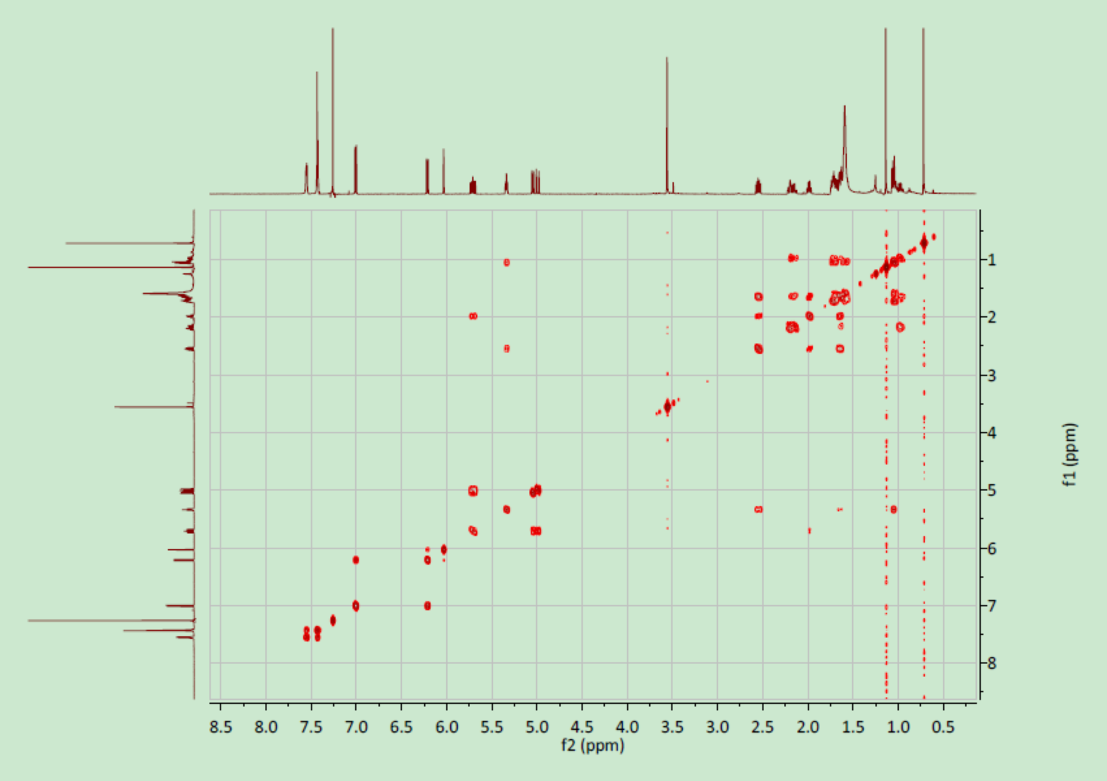

Figure S18. ESI-MS of compound **1s**.

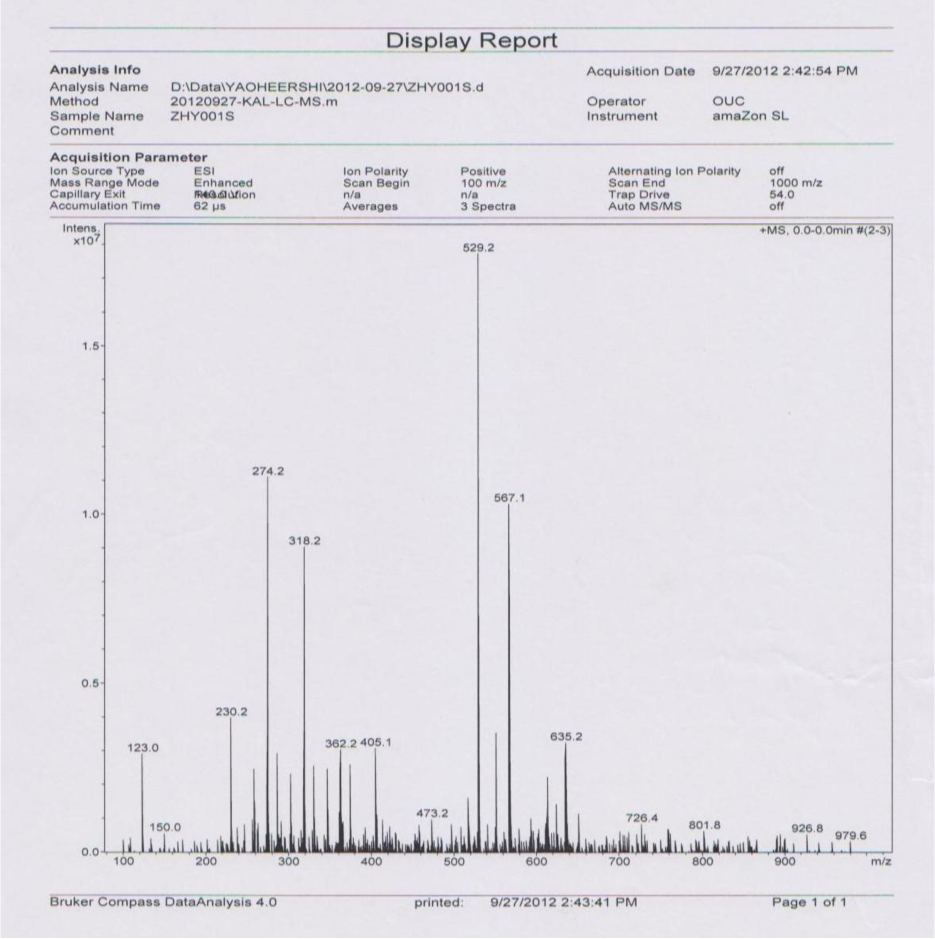

Figure S19. ^{1}H-NMR spectrum (600 MHz, $CDCl_3$) of compound **1r**.

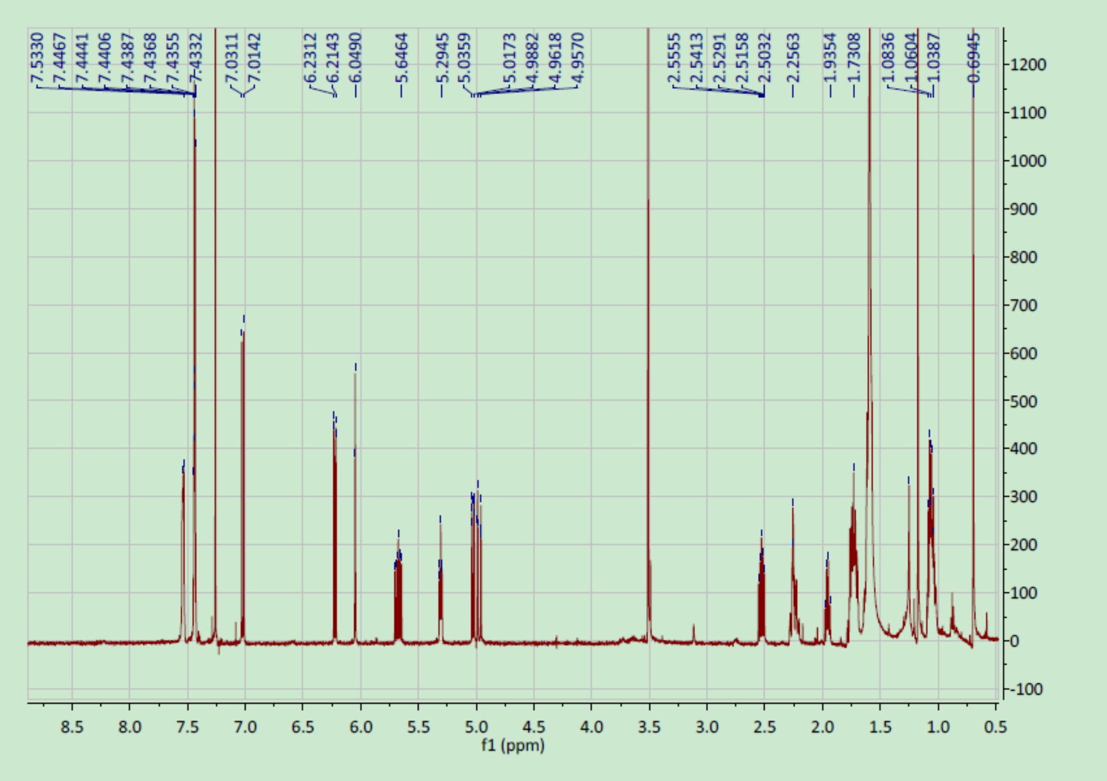

Figure S20. ^{1}H–^{1}H COSY spectrum of compound **1r** in $CDCl_3$.

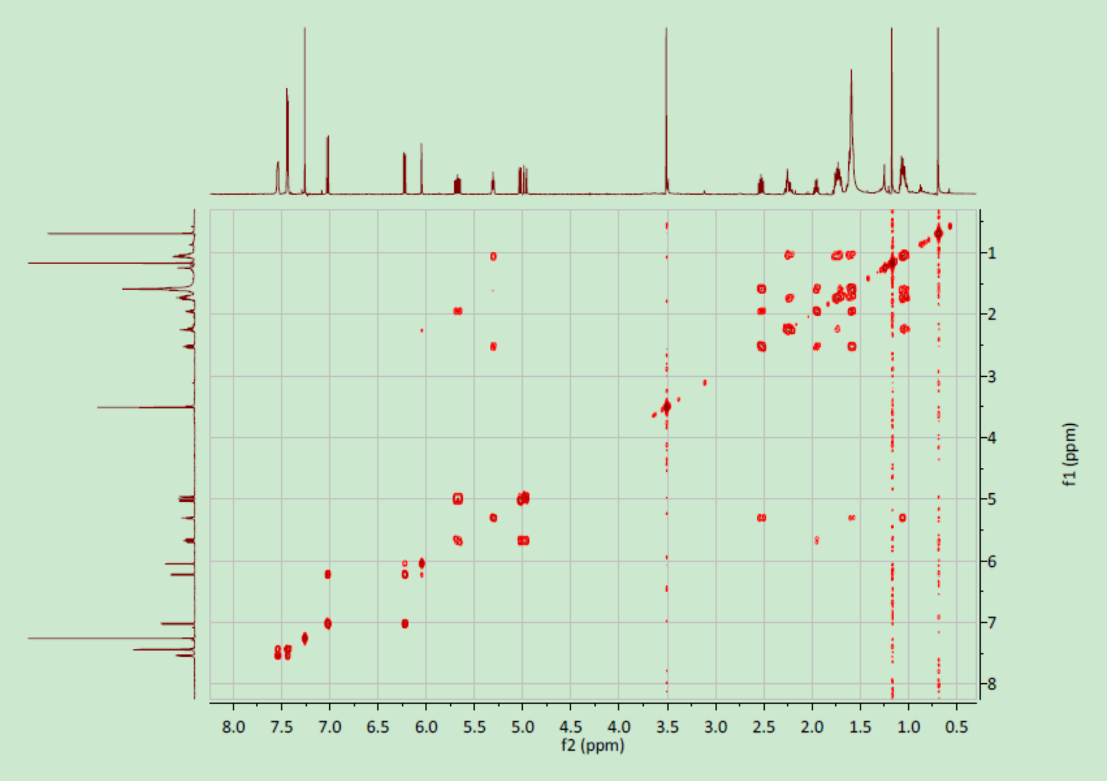

Figure S21. ESI-MS of compound **1r**.

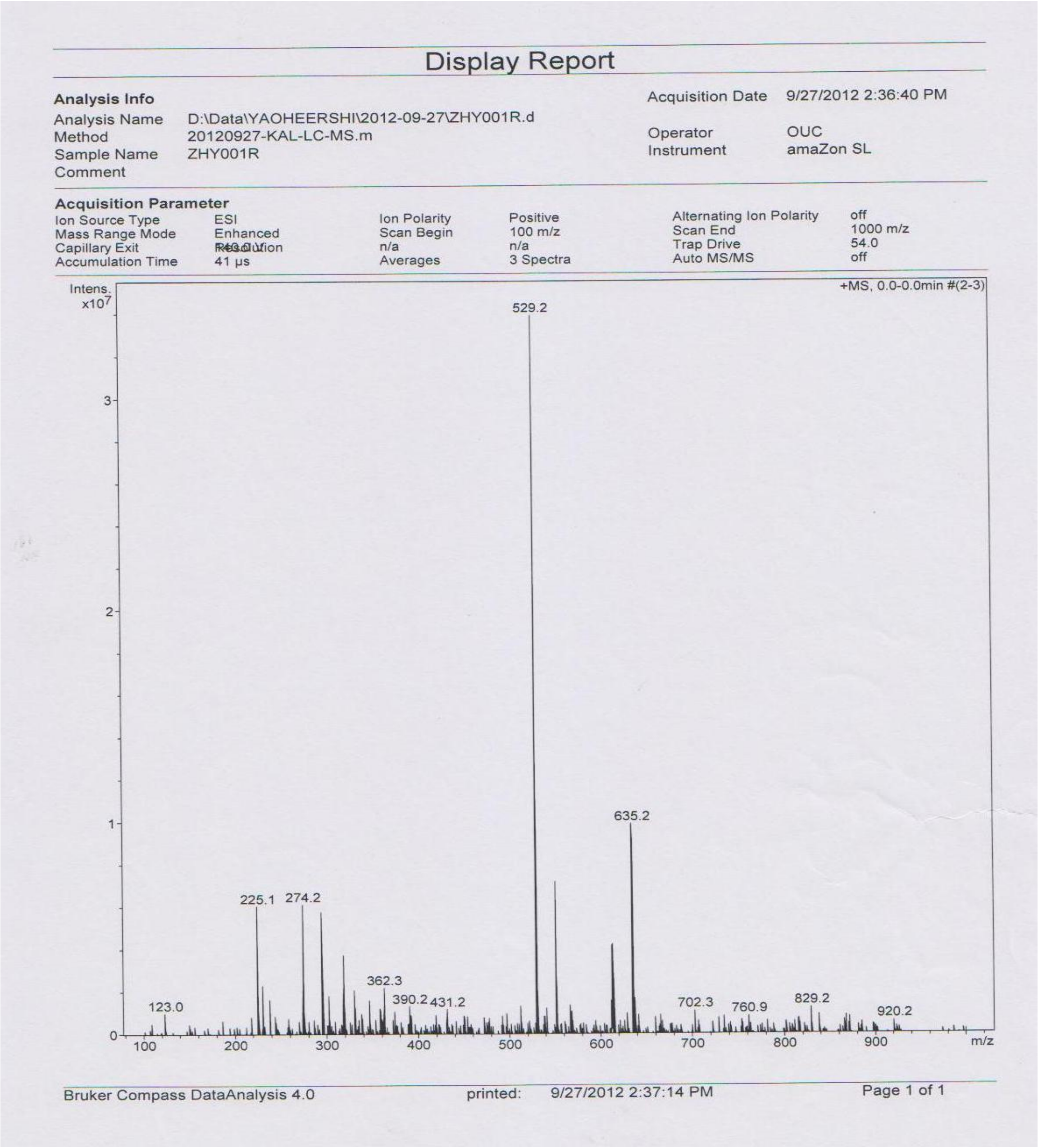

Supplement: Supplementary file 1 [file molecules-18-03458-s001.pdf]
